# Telemedical care and quality of life in patients with schizophrenia and bipolar disorder: results of a randomized controlled trial

**DOI:** 10.1186/s12888-021-03318-8

**Published:** 2021-06-29

**Authors:** Ulrike Stentzel, Neeltje van den Berg, Kilson Moon, Lara N. Schulze, Josephine Schulte, Jens M. Langosch, Wolfgang Hoffmann, Hans J. Grabe

**Affiliations:** 1 University Hospital Greifswald, Institute for Community Medicine, Section Epidemiology of Health Care and Community Health, Ellernholzstraße 1-2, 17487 Greifswald, Germany; 2 University Hospital Greifswald, Clinic and Polyclinic for Psychiatry and Psychotherapy, Greifswald, Germany; 3 Bethanien Hospital for Psychiatry, Psychosomatics and Psychotherapy, Greifswald, Germany

**Keywords:** Telemedical care, Telemedicine, Schizophrenia, Bipolar disorder, Quality of life, WHOQOL-BREF, Randomized controlled trial

## Abstract

**Background:**

Schizophrenia and bipolar disorder are serious psychiatric disorders with a high disease burden, a high number of years of life lived with disability and a high risk for relapses and re-hospitalizations. Besides, both diseases are often accompanied with a reduced quality of life (QoL). A low level of quality of life is one predictor for relapses. This study examines whether a telemedical care program can improve QoL.

**Methods:**

Post stationary **t**elem**e**di**c**a**l** c**a**re of patients with severe psychiatric disorders” (Tecla) is a prospective controlled randomized intervention trial to implement and evaluate a telemedical care concept for patients with schizophrenia and bipolar disorder. Participants were randomized to an intervention or a control group. The intervention group received telemedical care including regular, individualized telephone calls and SMS-messages. QoL was measured with the German version of the WHOQOL-BREF. Effects of telemedicine on QoL after 6 months and treatment*time interactions were calculated using linear regressions (GLM and linear mixed models).

**Results:**

One hundred eighteen participants were recruited, thereof 57.6% men (*n* = 68). Participants were on average 43 years old (SD 13). The treatment*time interaction was not significant. Hence, treatment had no significant effect either. Instead, gender is an influencing factor. Further analysis showed that social support, the GAF-level and QoL-values at baselines were significant determinants for the improvement of QoL.

**Conclusion:**

The telemedicine care concept Tecla was not significant for QoL in patients with severe psychiatric disorders. More important for the QoL is the general social support and the level of global functioning of the patients.

**Trial registration:**

German Clinical Trials Register, DRKS00008548, registered 21 May 2015 – retrospectively registered, https://www.drks.de/drks_web/setLocale_EN.do

**Supplementary Information:**

The online version contains supplementary material available at 10.1186/s12888-021-03318-8.

## Background

Mental disorders have a high disease burden and the number of days with limitations is 3 times higher in afflicted patients than for healthy people [1]. The course of mental diseases is often chronic [2]. Schizophrenia and bipolar disorder are among the most serious psychiatric disorders. Schizophrenia is one of the ten diseases with the highest number of years of life lived with disability (YLD) [3]. Relapses and re-hospitalization are frequent in patients with schizophrenia and bipolar disorder [4, 5]. Both diseases are often accompanied with a distinct impairment of social and professional life management and hence result in a lasting reduced quality of life [3, 6–8]. The World Health Organization Quality of Life (WHOQOL) Group defined quality of life as the “*individuals’ perceptions of their position in life in the context of the culture and value systems in which they live and in relation to their goals, expectations, standards and concerns*.” [9]. All aspects of life, which means physical, social, environmental and psychological aspects, affect one’s wellbeing and satisfaction [6].

Schizophrenia and bipolar disorder are both associated with poor quality of life [6]. The difference in quality of life of schizoaffective disorder is small compared to that of schizophrenia and bipolar disorder [10]. A low level of quality of life is a predictor for relapses [11]. Akvardar et al. showed that the improvement of quality of life is one important part in treating psychiatric disorders [7]. Hence, quality of life is an important factor and must be a target for gaining a good or at least stable state of mental health [7, 12].

Telemedicine has the potential to improve the health care situation for patients within the mental health spectrum. Positive effects were shown on patients with anxiety and depression [13] and on medication adherence in patients with schizophrenia and bipolar disorder [14].

This paper reports results regarding quality of life from a prospective controlled randomized intervention trial called “Post stationary **t**elem**e**di**c**a**l** c**a**re of patients with severe psychiatric disorders” (Tecla). Tecla’s objective was the implementation and evaluation of a telemedical care concept for patients with schizophrenia, schizoaffective disorder and bipolar disorder. It addressed different problematic issues in treatment and every-day-life-management [15]. Primary outcome was medication adherence, which was positively influenced by the telemedical care concept [14]. This article aims to investigate the effects of the telemedical care concept on the quality of life of patients with schizophrenia, schizoaffective disorder and bipolar disorder. The hypothesis is that the participants of the intervention group, which received additional telemedical care, had better levels of quality of life compared to participants of a control group, which received usual care six months after baseline.

## Methods

In this publication a secondary outcome of the Tecla study, quality of life, is reported. The primary outcome was medication adherence and is published elsewhere [14].

### Patient sample and data

Data were retrieved from the prospective pragmatic controlled randomized intervention trial Tecla. Tecla is a cooperation between the Institute for Community Medicine and the Department of Psychiatry and Psychotherapy, both University Medicine Greifswald, and the Bethanien Hospital for Psychiatry, Psychosomatics and Psychotherapy Greifswald gGmbH. An Integrated Telemedicine Centre (IFT) is affiliated to the Institute for Community Medicine [15, 16]. Inclusion criteria of Tecla were a medical diagnosis of any form of schizophrenia (ICD-10 F20), schizoaffective disorders (ICD-10 F25), or bipolar disorders (ICD-10 F31), and age ≥ 18 years. The approach was to evaluate the effectiveness of the intervention in real-life routine practice conditions. Hence, inclusion criteria were not further narrowed. The diagnoses were extracted from the patient files. Exclusion criteria were prior scheduled inpatient treatments within the next six months and lacking reachability by cell phone. The participants were recruited shortly before their discharge from day-care hospitals or open or locked inpatient wards from three psychiatric departments in the cities Stralsund and Greifswald (Western-Pomerania, a Federal State in the very northeast of Germany). Team members from the department of Psychiatry and Psychotherapy performed the recruitment and the baseline assessment. Personnel from the IFT conducted the telemedical care. A comprehensive description of the study protocol for the Tecla study was published by Stentzel et al. [15].

Tecla has been approved by the Ethics Committee of the University Medicine Greifswald (BB 122/14) and was registered at the German Clinical Trials Register (date 2015\05\21, DRKS00008548).

### Randomization

The participants were randomized to the intervention or control group after the baseline assessment. A blinded scientist, who was neither involved in the recruitment nor in the baseline assessment, performed the allocation to the groups using a random allocation (block randomization). The listing of the two groups was unregularly. The participants were chronically signed to the next entry in the randomization list.

### Telemedical intervention

**Fig. 1:**
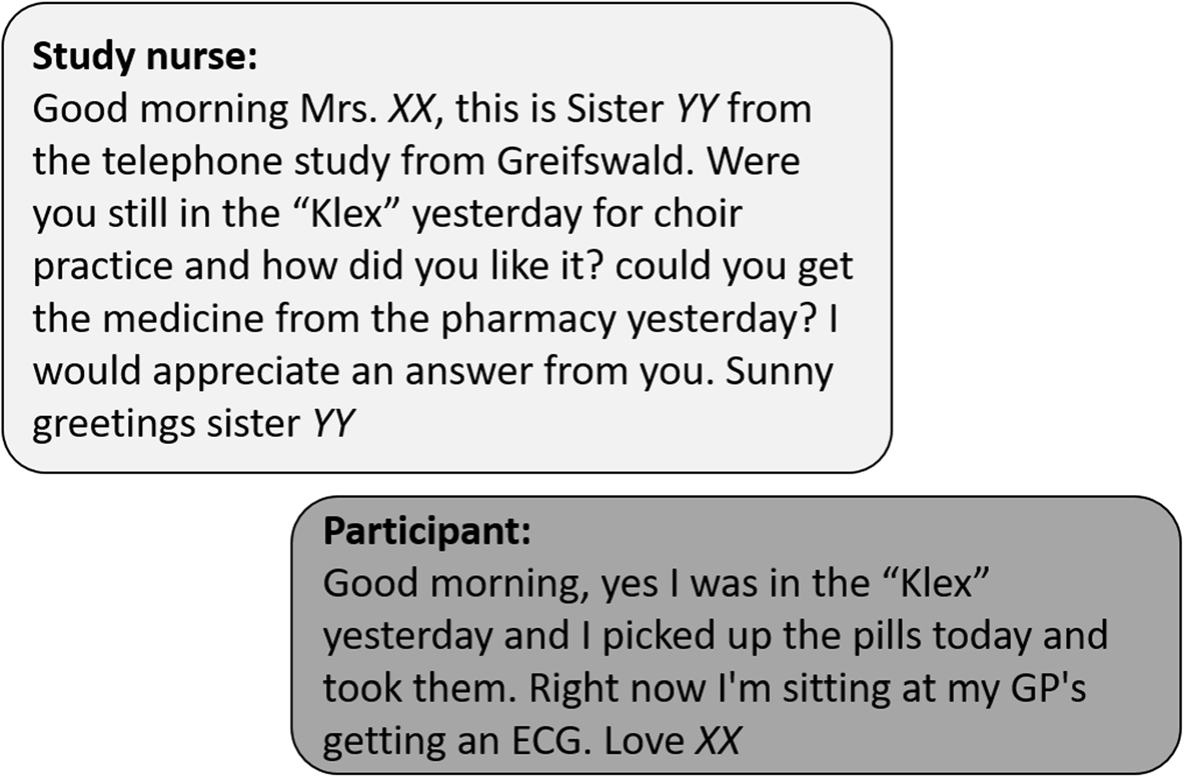
Example for an individualized text message contact between study nurse and participant

Participants were individually randomized to intervention group and control group. Both groups received care as usual in the outpatients facilities (outpatient psychiatric / psychotherapeutic practices or psychiatric institutional outpatients’ departments). The intervention group received regular telephone calls every two weeks and in addition standardized as well as individualized text messages every week. An example for such an individualized text message is given in Fig. 1. Qualified nurses who are specialized in telemedical care conducted the regular telephone calls. The nurses are embedded in regular meetings within one of the psychiatric institutional outpatients’ department and day-care hospital. They were trained in the documentation system and join appropriate psychiatric/psychotherapeutic education programs. The telemedical conversation was conducted on the basis of eCRFs in a computer-aided documentation system in accordance with the current standards for data security and data privacy [17, 18]. The standardized conversation contained a structured standardized and an individualized part. The structured standardized part of the telephone calls included suicidal tendencies, changes in the medication regime, medication adherence and medication side effects (study protocol published elsewhere [14]). The individualized part addressed selected topics of everyday life that the respective participant evaluated as important for himself and his condition. The weekly text messages refer to actual and relevant events and themes in the daily life of the participants.

### Measures

#### WHOQOL-BREF

The quality of life was measured with WHOQOL-BREF, the short version of the subjective instrument World Health Organization Quality of Life, which is designed for generic use [9, 19]. It assesses the quality of life from a subjective perspective [7]. The short version WHOQOL-BREF has 26 items. Answers are given on 1-to-5-point Likert scales.  The sum of all 26 items gives total quality of life, ranging from 26 to 130 [20]. The higher the score the better the quality of life of the patient [19]. WHOQOL assesses different aspects of life that are relevant for quality of life [9]. The WHOQOL-BREF bases on four domains [9, 19] and one global value for general quality of life:

- Physical domain: pain, energy, sleep, mobility, activities, medication, work.
- Psychological domain: positive feelings, cognitions, self-esteem, body image, negative feelings, spirituality.
- Social relationships domain: personal relationships, social support, sex.
- Environment domain: safety and security, home environment, finance, health/social care, information, leisure, physical environment, transport.
- Global value: overall quality of life, general health.

The German version was used, which shows good internal consistence (Cronbachs α > 0.7 for all domains) for the overall population as well as for patients with mental disorders [21].

#### Social support

Social support was assessed using the measure F-SozU (Social support, short form with 14 items) [22]. The authors defined social support as the result of cognitive-emotional processing and assessment of current and past social interactions. The concept is based on cognitive approaches and assesses the subjective conviction to get support from the subject’s social network if necessary. This 14-item short form is appropriate for the assessment of a more generally perceived social support [22]. The statements refer to the fields of emotional support (to be liked and accepted by others, to share feelings, to experience participation), to provide practical assistance (practical help in everyday problems, for example to borrow things, getting practical advice, getting help with challenging tasks) and social integration (belonging to a circle of friends, doing joint ventures, knowing people with similar interests) and are assessed using a 5 category Likert-scale from “does not apply” (scored 1) to “applies exactly” (scored 5) [22, 23].

#### Global assessment of functioning (GAF)

The Global Assessment of Functioning (GAF) is an overall measure of how patients are doing from positive mental health up to severe psychopathology [24]. It is known, that functioning is low in people with current mental health disorders, so functioning can be used as an expression of the severity of illness [25]. The GAF-questionnaire measures the degree of mental illness by rating psychological, social and occupational functioning [24] on an ordinal scale from 1 to 100 [26]. The scale is divided into 10-point intervals. The lowest interval (score 1 to 10) represents severe illness, the highest interval (score 91 to 100) represents the healthiest condition [23, 24].

#### Participants’ evaluation of the telemedical care program

Participants of the intervention group were asked to evaluate the telemedical care at the end of their study participation by answering the questions shown in Table 1.

**Table 1:** Interview questions and answers to assess acceptance and satisfaction of the participants

Question:	How would you assess the telephone and text messages contacts during the last 6 months?
Answer:	Very helpful – little helpful – not helpful – other (free text) – don’t know – no answer
Question:	Could you imagine continuing the telephone contacts in this form?
Answer:	Yes – No – don’t know – no answer
Question:	Do you think this kind of care can partly replace personal contacts with physicians or psychologists?
Answer:	Yes – No – don’t know – no answer
Question:	Is there something you would change or improve?
Answer:	Yes – No – don’t know – no answer and additional free text

### Statistical analysis

The baseline characteristics were compared by group affiliation to identify any group differences at baseline. Linear mixed models were calculated to test for the intervention effects and for treatment*time interaction for WHOQOL total quality of life and all WHOQOL domains. The computation was performed using SAS PROC MIXED (SAS 9.4© 2002–2012 by SAS Institute Inc., Cary, North Carolina, USA.). For parameter estimation, a minimum variance quadratic unbiased estimation (MIVQUE0) was performed, using unstructured covariance matrices. The WHOQOL total quality of life as well as each of the WHOQOL domains and the global value were the respective dependent variable. As fixed effects served the affiliation to the patient group, age, gender and education. A treatment*time interaction was included. A further set of models was calculated with the variables social support and GAF besides to the previously used. Furthermore, a generalized linear regression was calculated to analyze the change of quality of life at the six-month-follow-up compared to the quality of life value at baseline. Results are considered statistically significant when p-values are 0.05.

The analyses were conducted with the intention-to-treat approach. For randomized clinical trials with missing data the multiple imputation procedure is a valid method to handle missing data [27] and to minimize possible biases [28]. However, a required condition for multiple imputation is, that missing data are distributed completely at random (MCAR) or at random (MAR), whereas the method is less appropriate for data missing not at random (MNAR) [29]. After thorough inspection, we appraised the missing data as MAR. The proportions of missing values ranged from 11 to 17% (WHOQOL-variables 12%). Hence multiple imputation was proceeded. To be able to reproduce the results, each time the analysis is performed the random seed value was specified [27]. Eighteen variables were included in the imputation model. Minimum and maximum values for score values were defined. Further details are documented in the supplement. All statistical procedures were performed in SAS 9.4 (© 2002–2012 by SAS Institute Inc., Cary, North Carolina, USA.) with the procedure PROC MI and PROC MIANALYZE.

## Results

**Fig. 2:**
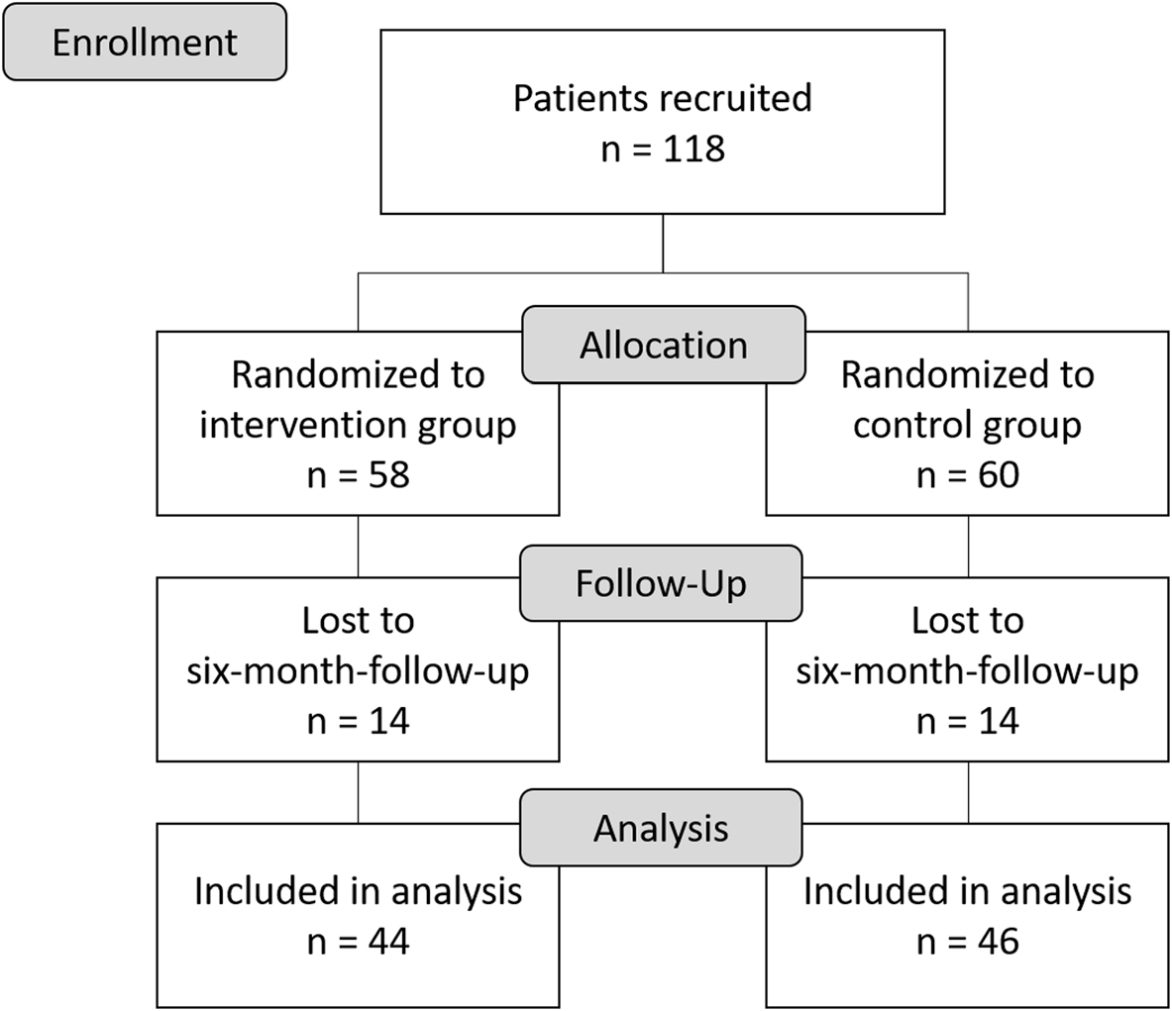
CONSORT flow diagram [30]

118 participants were recruited (see CONSORT flow diagram in Fig. 2), thereof 57.6% men (*n* = 68). Participants were on average 43 years old (standard deviation (SD) 13). Baseline characteristics are shown in Table 2. Except for education, there was no significant difference between the intervention and control group at baseline. Participants in the intervention group had a better education than participants in the control group. 104 diagnoses of schizophrenia and schizoaffective disorder (ICD-10 F2x.) and 48 bipolar disorder-diagnoses (ICD-10 F3x.) were found. 21 patients had two to three diagnoses. Further details are documented in Table 1 in the supplement. 90 participants remained in the study until the six-month-follow-up. Of these, 79 participants completed the WHOQOL-BREF.

**Table 2:** Characteristics of the participants at baseline. The differences between the intervention and control group were analyzed for categorical variables with Chi^2^ and for continuous variables with a t-test

**Chi**^**2**^	**Total** **n (%)**	**Intervention group** **n (%)**	**Control group** **n (%)**	**p-value**
Participants	118 (100)	58 (49.2)	60 (50.8)	
Female	50 (42.4)	27 (22.9)	23 (19.5)	0.3664
Psychiatric disease^a^				0.4734
Schizophrenia / Schizoaffec-tive disorder (ICD-10 F2x.)	104 (68.4)	52 (34.2)	52 (34.2)	
Bipolar disorder (ICD-10 F3x.)	48 (31.6)	21 (13.8)	27 (17.8)	
Education:				0.0002
< 10 years	32 (32.3)	6 (6.1)	26 (26.3)	
10 years	42 (42.4)	25 (25.3)	17 (17.2)	
> 10 years	25 (25.3)	17 (17.2)	8 (8.1)	
Employment:				0.3483
Not employed	98 (85.2)	45 (39.1)	53 (46.1)	
Marginally employed	5 (4.4)	3 (2.6)	2 (1.7)	
Employed	12 (10.4)	8 (7.0)	4 (3.5)	
Social living situation:				0.9299
Living alone	56 (51.4)	27 (24.8)	29 (26.6)	
Living with spouse, partner or assisted living	53 (48.6)	26 (23.9)	27 (24.8)	
**t-test**	**Total** **mean (SD)**	**Intervention group** **mean (95% CI)**	**Control group** **mean (95% CI)**	**p-value**
age	42.9 (13.0)	43.9 (40.5–47.4)	42.0 (38.6–45.2)	0.4099
Social support	48.9 (13.1)	48.8 (10.8–15.8)	48.9 (11.3–16.6)	0.9480
GAF	55.3 (11.0)	55.5 (9.1–13.2)	55.2 (9.7–14.0)	0.8951
WHOQOL total quality of life	87.2 (14.0)	86.8 (83.0–90.8)	87.6 (83.6–91.2)	0.7927
WHOQOL domains:				
Global	49.3 (21.0)	46.0 (39.9–52.2)	52.4 (46.8–58.0)	0.1246
Physical health	56.3 (16.7)	56.8 (51.9–61.5)	55.8 (51.2–60.5)	0.7931
Psychological	56.3 (17.2)	56.8 (51.9–61.7)	55.8 (51.1–60.6)	0.7740
Social relationships	57.3 (21.3)	53.7 (48.0–59.3)	60.7 (54.5–66.9)	0.0938
Environment	66.1 (15.2)	66.3 (62.2–70.5)	65.9 (61.5–70.3)	0.8826

^a^Higher overall numbers because some patients had both diagnoses, *CI* confidence interval, *GAF* Global Assessment of Functioning

The treatment*time interaction was not significant for either the WHOQOL total quality of life nor for the WHOQOL domains and the global value. *P*-values were ranging between 0.123 to 0.519. The further results of the linear mixed model regressions are shown in Table 3. A significant influencing factor is the participants’ gender. Being male showed higher values for the WHOQOL total quality of life score and all domains except for the social relationships. Age showed significant results regarding the domains social relationships and environment. The estimate ranged between 0.20 to 2.7 though. To control for the observed differences at baseline, the level of education (< 10 years, 10 years, > 10 years) was included in the model. Except the domain environment, education showed no significant results. Regarding environment, higher education (> 10 years) showed higher values of quality of life.

**Table 3:** Results of the linear mixed model for WHOQOL total quality of life and the five WHOQOL Domains

WHOQOL		Estimate	95% CI		p-value^a^
			LCI	UCI	
**Total score quality of life**					
Intercept		76.677	67.927	85.427	**<.0001**
study group (ref = control)		1.119	−4.231	6.469	0.682
time point (ref = baseline)		0.980	− 4.528	6.489	0.727
age		0.120	−0.047	0.287	0.159
gender (ref = female)		8.669	4.597	12.741	**<.0001**
education (ref = < 10 years)	10 years	−1.279	−6.256	3.698	0.614
	> 10 years	3.066	−2.828	8.960	0.307
study group * time point		4.740	−2.995	12.475	0.230
**Physical health domain**					
Intercept		49.469	38.878	60.061	**<.0001**
study group (ref = control)		1.775	−4.817	8.366	0.598
time point (ref = baseline)		1.741	−5.078	8.561	0.617
age		0.032	−0.171	0.235	0.757
gender (ref = female)		7.784	2.831	12.736	**0.002**
education (ref = < 10 years)	10 years	−0.936	−7.191	5.320	0.769
	> 10 years	5.358	−1.897	12.614	0.148
study group * time point		4.424	−5.089	13.938	0.362
**Psychological health domain**					
Intercept		47.082	35.691	58.472	**<.0001**
study group (ref = control)		3.134	−3.888	10.156	0.382
time point (ref = baseline)		1.741	−5.078	8.561	0.934
age		0.039	−0.181	0.258	0.730
gender (ref = female)		12.472	7.103	17.842	**<.0001**
education (ref = < 10 years)	10 years	−3.517	−10.087	3.054	0.294
	> 10 years	0.647	−7.037	8.331	0.869
study group * time point		4.432	−5.756	14.619	0.394
**Social relationships domain**					
Intercept		47.436	33.747	61.125	**<.0001**
study group (ref = control)		−2.840	−11.082	5.402	0.499
time point (ref = baseline)		−0.490	−8.984	8.004	0.910
age		0.274	0.018	0.531	**0.036**
gender (ref = female)		3.875	−2.406	10.157	0.226
education (ref = < 10 years)	10 years	−4.257	−11.977	3.463	0.280
	> 10 years	−5.376	− 14.399	3.647	0.243
study group * time point		3.976	−8.116	16.067	0.519
**Environment domain**					
Intercept		51.136	41.765	60.508	**<.0001**
study group (ref = control)		1.462	−4.304	7.228	0.619
time point (ref = baseline)		0.777	−4.986	6.541	0.791
age		0.200	0.025	0.375	**0.025**
gender (ref = female)		7.054	2.818	11.29	**0.001**
education (ref = < 10 years)	10 years	0.997	−4.200	6.195	0.707
	> 10 years	6.602	0.323	12.88	**0.039**
study group * time point		3.814	−4.333	11.962	0.359
**Global Domain**					
Intercept		43.37	30.463	56.278	**<.0001**
study group (ref = control)		−3.211	−11.288	4.867	0.436
time point (ref = baseline)		4.694	−3.661	13.049	0.271
age		0.060	−0.192	0.312	0.642
gender (ref = female)		9.679	3.625	15.733	**0.002**
education (ref = < 10 years)	10 years	0.233	−7.142	7.608	0.951
	> 10 years	−0.706	−9.446	8.033	0.874
study group * time point		9.245	−2.496	20.985	0.123

*Abbreviations*: *WHOQOL* World Health Organization Quality of Life, *CI* confidence interval, *LCI* lower CI (mean), *UCI* upper CI (mean)

^a^significant *p* values are printed in bold

Table 4 shows the results of the further set of calculated models were the additional variables social support and GAF were included. Again, treatment*time interactions were not significant, accordingly treatment had no effect on the quality of life. Gender shows very similar results as in the models with less variables. Being male again showed significantly higher values for the WHOQOL total quality of life score, the global value and all domains except for the social relationships. Increasing social support showed significantly increasing values for WHOQOL total quality of life score, the psychological domain, social relationships, environment and the global value (estimates ranges from 0.27 to 0.82 though).

**Table 4:** Results of the linear mixed model for WHOQOL total sum score and the five WHOQOL Domains with further variables

WHOQOL		Estimate	95% CI		p-value^a^
			LCI	UCI	
**Total score quality of life**					
Intercept		46,772	35,965	57,579	**<.0001**
study group (ref = control)		1752	− 2869	6373	0,457
time point (ref = baseline)		− 4293	− 9640	1053	0,115
age		− 4293	−0,114	0,175	0,676
gender (ref = female)		7044	3493	10,595	**<.0001**
education (ref = < 10 years)	10 years	− 4047	− 8481	0,388	0,074
	> 10 years	− 2957	− 8412	2499	0,287
Social support		0,371	0,223	0,519	**<.0001**
GAF		0,345	0,183	0,508	**<.0001**
study group * time point		3.769	−2.966	10.504	0.273
**Physical health domain**					
Intercept		22,183	7678	36,688	**0,003**
study group (ref = control)		2626	− 3504	8756	0,401
time point (ref = baseline)		− 5176	−12,140	1788	0,145
age		−0,038	−0,229	0,152	0,692
gender (ref = female)		6286	1665	10,907	**0,008**
education (ref = < 10 years)	10 years	− 3691	− 9562	2179	0,217
	> 10 years	− 0,635	−7,77	6,5	0,861
Social support		0,142	−0,05	0,333	0,147
GAF		0,481	0,265	0,697	**<.0001**
study group * time point		3.100	−5.804	12.005	0.495
**Psychological health domain**					
Intercept		14,726	−0,408	29,860	0,056
study group (ref = control)		3937	− 2472	10,346	0,229
time point (ref = baseline)		− 6867	−14,281	0,547	0,069
age		−0,053	−0,256	0,149	0,606
gender (ref = female)		10,707	5728	15,687	**<.0001**
education (ref = < 10 years)	10 years	− 6610	−12,831	− 0,390	**0,037**
	> 10 years	− 6086	− 13,589	1416	0,112
Social support		0,322	0,112	0,532	**0,003**
GAF		0,441	0,206	0,676	**<.0001**
study group * time point		3.212	−6.117	12.541	0.500
**Social relationships domain**					
Intercept		2867	−13,838	19,572	0,736
study group (ref = control)		− 2272	− 9210	4666	0,521
time point (ref = baseline)		− 5500	− 13,558	2559	0,181
age		0,126	− 0,086	0,338	0,244
gender (ref = female)		1465	− 3840	6771	0,588
education (ref = < 10 years)	10 years	− 8081	− 14,701	− 1462	**0,017**
	> 10 years	−13,705	−21,747	− 5662	**0,001**
Social support		0,821	0,035	1038	**<.0001**
GAF		0,289	0,035	0,544	**0,026**
study group * time point		3.122	−7.101	13.345	0.549
**Environment domain**					
Intercept		30,174	17,934	42,415	**<.0001**
study group (ref = control)		1685	− 3703	7074	0,540
time point (ref = baseline)		− 1219	− 7302	4863	0,694
age		0,129	−0,033	0,291	0,119
gender (ref = female)		5929	2010	9848	**0,003**
education (ref = < 10 years)	10 years	− 0,754	− 5761	4252	0,767
	> 10 years	2793	− 3612	9198	0,392
Social support		0,418	0,246	0,591	**<.0001**
GAF		0,109	−0,074	0,291	0,243
study group * time point		3.479	−4.071	11.030	0.366
**Global Domain**					
Intercept		12,838	− 4603	30,278	0,149
study group (ref = control)		− 2434	−10,072	5204	0,532
time point (ref = baseline)		− 1822	−10,631	6986	0,685
age		− 0,025	−0,267	0,217	0,838
gender (ref = female)		8016	2265	13,766	**0,006**
education (ref = < 10 years)	10 years	− 2693	− 9726	4339	0,453
	> 10 years	− 7089	−15,768	1590	0,109
Social support		0,272	0,025	0,519	**0,031**
GAF		0,443	0,181	0,705	**0,001**
study group * time point		8.025	−3.091	19.142	0.157

*Abbreviations*: *WHOQOL* World Health Organization Quality of Life, *CI* confidence interval, *LCI* lower CI (mean), *UCI* upper CI (mean), *GAF* Global Assessment of Functioning

^a^significant p values are printed in bold

With increasing level of the Global Assessment of functioning as a measure for the impairment of the participants, the WHOQOL total quality of life, the physical, psychological and social relationships domain and the global value increased significantly. The increasing ranges from 0.29 to 0.48 points though. Education showed different results as in the first calculated models. Here, education became more significant regarding the psychological domain and the social relationships domain. Psychological domain: quality of life decreased with 10 years of education significantly, more than 10 years was not significant. Social relationships domain: quality of life decreased with both 10 years and more than 10 years of education significantly. Whereas in the environment domain education was not significant.

The results regarding the change of quality of life at six-month-follow-up compared to baseline, calculated with generalized linear regression models, are shown in Table 5. Similarly, in these calculations allocation to the intervention or control group is not significant. In contrast to previous models, however, gender is not significant. Highly significant for the change of WHOQOL total quality of life score, all domains and the global value (comparing 6-month-follow-up with baseline values), was the quality of life value at baseline. With increasing WHOQOL value at baseline, the change between 6-month follow-up and baseline gets smaller. The factor ranges between − 0.23 to − 0.66.

**Table 5:** Results of the generalized linear model for the change (six-month-follow-up compared with baseline) of the WHOQOL total sum score and the five WHOQOL Domains

WHOQOL		Estimate	95% CI		***p***-value^a^
			LCI	UCI	
**Total score quality of life**					
Intercept		61,914	43,318	80,510	**<.0001**
study group (ref = control)		4348	−0,951	9648	0,108
age		−0,049	− 0,259	0,161	0,648
gender (ref = female)		− 5213	−10,575	0,148	0,057
education (ref = < 10 years)	10 years	2143	− 3888	8173	0,486
	> 10 years	0,305	− 7168	7778	0,936
BL-Total Score^b^		−0,662	− 0,856	− 0,468	**<.0001**
**Physical health domain**					
Intercept		32,260	18,319	46,201	**<.0001**
study group (ref = control)		3787	− 1958	9531	0,196
age		−0,135	− 0,364	0,093	0,245
gender (ref = female)		− 2566	− 8397	3265	0,387
education (ref = < 10 years)	10 years	2474	− 4079	9026	0,459
	> 10 years	0,494	− 7622	8611	0,905
BL-Total Score		−0,462	− 0,641	− 0,283	**<.0001**
**Psychological health domain**					
Intercept		31,005	16,921	45,088	**<.0001**
study group (ref = control)		4318	− 1498	10,133	0,145
age		−0,145	−0,376	0,086	0,219
gender (ref = female)		− 4474	−10,324	1375	0,134
education (ref = < 10 years)	10 years	2071	− 4637	8778	0,545
	> 10 years	− 2354	− 10,629	5921	0,576
BL-Total Score		− 0,408	−0,568	− 0,249	**<.0001**
**Social relationships domain**					
Intercept		18,092	5127	31,056	**0,006**
study group (ref = control)		1174	− 5078	7426	0,713
age		−0,099	− 0,375	0,178	0,483
gender (ref = female)		0,638	− 5643	6920	0,842
education (ref = < 10 years)	10 years	3119	− 4485	10,724	0,421
	> 10 years	− 4002	−13,419	5415	0,403
BL-Total Score		− 0,227	−0,372	− 0,082	**0,002**
**Environment domain**					
Intercept		28,289	12,585	43,992	**<.0001**
study group (ref = control)		2710	− 3648	9068	0,403
age		−0,088	−0,353	0,178	0,516
gender (ref = female)		− 1515	− 8029	5000	0,648
education (ref = < 10 years)	10 years	5183	− 1995	12,361	0,157
	> 10 years	1257	− 7989	10,503	0,789
BL-Total Score		−0,378	− 0,591	− 0,166	**0,001**
**Global Domain**					
Intercept		23,199	11,038	35,359	**<.0001**
study group (ref = control)		0,602	11,038	6466	0,840
age		−0,110	−0,348	0,128	0,363
gender (ref = female)		− 2465	− 8339	3409	0,410
education (ref = < 10 years)	10 years	4572	− 2143	11,287	0,182
	> 10 years	0,229	− 8452	8909	0,959
BL-Total Score		− 0,344	−0,487	− 0,202	**<.0001**

*Abbreviations*: *WHOQOL* World Health Organization Quality of Life, *CI* confidence interval, *LCI* lower CI (mean), *UCI* upper CI (mean)

^a^significant p values are printed in bold

^b^Baseline WHOQOL Total Score value

**Fig. 3:**
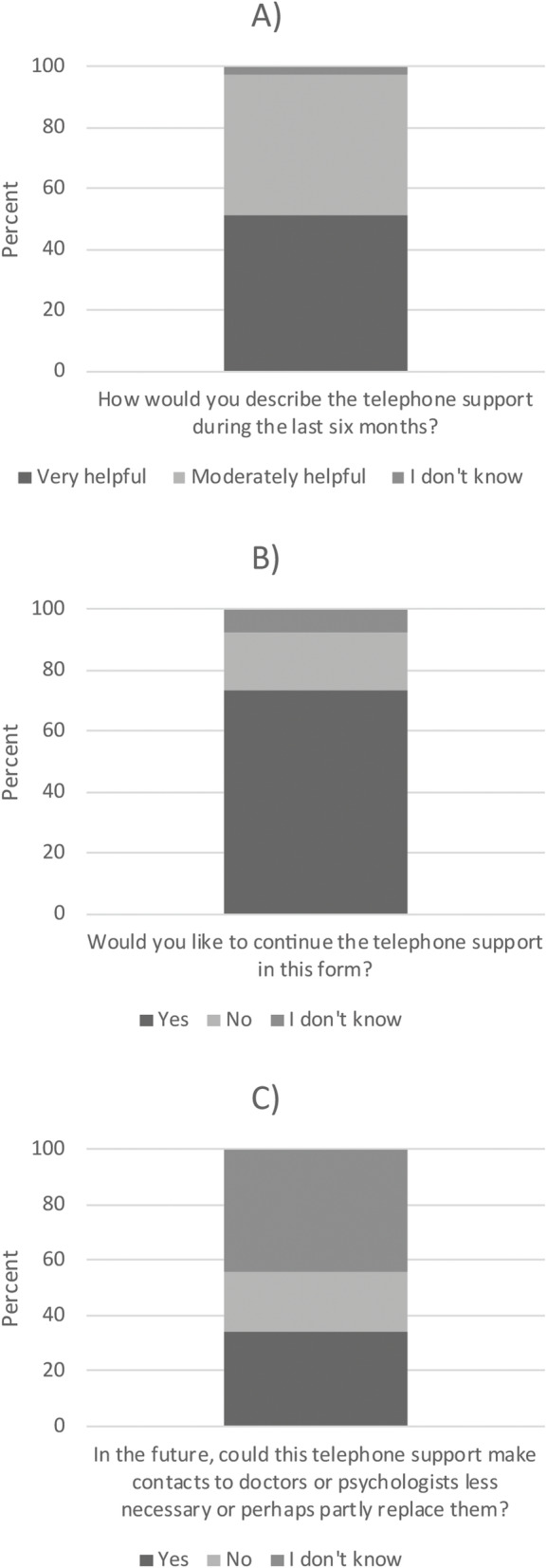
Subjective evaluation of received telemedicine care by the participants of the intervention group

The results of the evaluation of the telemedical program by participants of the intervention-group are shown in Fig. 3. Participants perceived the telemedical care mostly as moderately to very helpful (97.5%, Fig. 3A). A majority would like to continue the telemedical care (73.2%, Fig. 3B). A minority can even imagine, that the tele medical care can make contacts to doctors or psychologists less necessary or perhaps can partly replace them (34.2%, Fig. 3C).

## Discussion

Quality of life is a major treatment goal for patients with psychiatric disorders [7, 12, 31]. Quality of life was a secondary outcome in this study. The results of primary outcome medication adherence is published elsewhere [14]. Authors assumed that telemedicine care may have a positive influence not only on medication adherence, but also on quality of life. These expectations have not been confirmed. This may be due to the fact that as a secondary outcome the focus of the telemedical care was not primarily on quality of life but on medication adherence. An Israeli study investigated a mobile health (mHealth) approach. Ben-Zeev (et. al) compared the mHealth intervention FOCUS with a widely used group self-management intervention called WRAP [32]. As one of the secondary outcomes quality of life was investigated. Contrary to our findings the FOCUS participants showed significant improvements between baseline and the six-months-follow-up. Even though the FOCUS intervention substantially differs slightly from Tecla, the mode of administration via information and communication technologies is similar. The general feasibility, acceptance and efficiency of electronic Health (eHealth) and mHealth interventions for people with serious mental illnesses is proven by several other studies [33–35].

As influencing factors age, gender, the education level, social support and the global functioning level (GAF) were revealed int linear mixed models. Age is known to be significantly related to quality of lives in patients with schizophrenia [36]. Although age was occasionally significant, the estimates are very low and are all close to zero. Compared to all factors gender (being male) showed the strongest influence in the linear mixed models. The results regarding age and male gender are corresponding with other studies [31]. Where education showed significant influence, the observed estimates were moderate. Some authors regard the relationship between socio-demographic factors and quality of life as controversial, weak, or non-existent [37, 38], but some reported significant associations [39, 40]. Our results vary and do not clearly support either view. Social support has a known positive influence on quality of life [38, 41, 42]. This was also significantly verified in our results. The improvement amounted moderate 0.27 to 0.82 points though. To consider also the by the disease caused disability of the subjects the GAF was included in the model. Corresponding to other studies [38, 40, 43], higher GAF levels showed significant better quality of life levels for all domains and the WHOQOL total quality of life. Similarly here, too, the estimates increased by merely moderate values (from 0.29 to 0.48 points). The generalized linear regression models revealed that the change between six-month-follow-up compared to the baseline values decreased with increasing baseline values. This is corresponding to the findings regarding GAF. The better the global functioning level and the higher the quality of life values at the end of an acute inpatient hospital stay, the more likely is an improvement of quality of life afterwards.

However, the WHOQOL was proven as an adequate tool for assessing quality of life in different cultures and population groups [44, 45]. Therefore, in this study we have adopted a generic tool [6], that can be broadly applied for assessing quality of life in different cultures and population groups [46, 47]. The WHOQOL-BREF is less affected by disease-related factors [19] and has been applied in patients with schizophrenia with good reliability and validity [38, 47], even in psychotic stages, on medication and in patients with relatively low education level [7]. Kim et al. compared patients’ assessments of their own quality of life with WHOQOL-BREF with assessments of proxies (such as family members, caregivers) and found a moderate to good accordance between both assessments of the patients’ quality of life [8].

Even though schizophrenia and bipolar disorder are different diseases, there are similarities between them like the extent of quality of life. Both diseases showed similar scores for the WHOQOL-BREF domains in previous studies [12, 48]. In this study, the baseline characteristics showed no differences between the diagnostic groups (see Table 2). Hence, we analyzed both diseases together.

A strength of this study is the usual care setting with only little inclusion and exclusion criteria. Consequently, the results are likely to be transferable to a large part of the patient group and daily regular medical care. In this regular care setting, the study was conducted with a pragmatic RCT-design. To fortify the validity, a multiple imputation was performed.

The baseline assessment showed a significant difference between the two groups with respect to the level of education. Participants in the intervention group had a higher level of education compared to participants in the control group. A blinded scientist performed the allocation to the groups using a random allocation (block randomization) after the baseline assessment. However, the baseline characteristics showed similar values for all WHOQOL-domains for both groups (see Table 2). In fact, the intervention group had even slightly lower WHOQOL total score values. The intervention was largely standardized. Furthermore, the loss to follow-up was identical in both groups (see Fig. 2). Therefore, a systematic bias seems unlikely. The proportion of loss to follow-up at the six-month-follow-up was 24% in the invention group and 23% in the control group. Due to the size of the dropout rates, there might be an attrition bias [49, 50], but threshold levels for acceptable dropout-levels are not determined in guidelines yet [50]. Furthermore, distinct patient clienteles might require different levels. Because of the almost identical rates and because of the difficult patient clientele, we deem that potentially bias might be low. Besides, the loss to follow-up is similar to other reported dropout rates in the regarded patient groups [47]. To consider this fact, education was included in the model to control for it.

Diagnoses were extracted from the patients’ files from the three recruiting psychiatric departments. This could be a potential source of selection bias. In several cases, a clear diagnosis has not yet been made by the treating physicians. Therefore sometimes several diagnoses were applied here.

The duration of the illness is considered as important factor in the literature [37]. In the Tecla study, it was gathered from the patients records by date of first diagnosis. The date was more often not available than available so that it was not possible to include the duration of the illness in to the model.

Medication and its side effects could possibly affect patients’ quality of life [12] and would have been informative, but these aspects were not included here. However, it is a relevant question. Hence, the influence of medication on various data collected within the Tecla study, including the quality of life aspect, is currently being evaluated.

## Conclusion

Every aspect that can help stabilize the patient and avoid hospitalization should be considered in the treatment. The telemedicine intervention shown here is a low-threshold care concept that has the potential to improve the care situation of patients with severe psychiatric illness. Schulze et al. previously showed that Tecla improved medication adherence [14]. The intervention was successfully transferred to standard care. Here, we examined the impact of Tecla on participants’ quality of life. Quality of life concerns the personal, subjective perspective of life and has a high relevance for patients. The telemedicine care intervention Tecla addressed both general and individual issues of the participants’ daily life. However, the focus was primarily on medication adherence.

## Supplementary Information

**Additional file 1.**

## Data Availability

The datasets used and/or analysed during the current study are available from the corresponding author on reasonable request.

## Abbreviations

BL: Baseline
CI: Confidence interval
eCRF: Electronic Case Report Forms
eHealth: Electronic health
F-SozU: Questionnaire for Social Support
GAF: Global Assessment of Functioning
GLM: Generalized linear model
LCI: Lower confidence interval
MAR: Missing at random
MCAR: Missing completely at random
MNAR: Missing not at random
mHealth: Mobile health
MI: Multiple imputation
QoL: Quality of Life
SD: Standard deviation
Tecla: Study “Post stationary **te**lemedi**c**a**l** c**a**re of patients with severe psychiatric disorders”
UCI: Upper confidence interval
WHOQOL: World Health Organization Quality of Life
WHOQOL-BREF: World Health Organization Quality of Life, short form with 26 items
YLD: Years of life lived with disability
