# Supplementary material for: Telemedical care and quality of life in patients with schizophrenia and bipolar disorder: results of a randomized controlled trial
Source: BMC Psychiatry. 2021 Jun 29;21:318. doi: 10.1186/s12888-021-03318-8 (PMC8243575; doi:10.1186/s12888-021-03318-8)
Supplement: Supplementary file 1 — Additional file 1. [file 12888_2021_3318_MOESM1_ESM.docx]

# Supplement: Multiple imputation

It is valid to ignore the missing data if the missing data is less than 5 % [1]. Multiple imputation is appropriate when the proportion of missing data is not too large (more than 40 %) [1]. In this study, the quality of life data showed 12 % missing data at six-month-follow-up. The range of missing data for all variables that were included in the multiple imputation procedure amounts 11 – 17 %. Hence, the missing data is within the range that allows multiple imputation. Besides the lost-to-follow-up from baseline to six-month-follow-up was 24 %. The missing data causes a loss of power (and hence a higher risk for beta errors) which is also an indication for multiple imputation [2]. The multiple imputation was performed with the SAS procedure PROC MI in SAS 9.4 (© 2002-2012 by SAS Institute Inc., Cary, North Carolina, USA.). The PROC MI results revealed a non-monotone arbitrary missing pattern. The number of imputations was set at 20. According to literature, the number of imputations should be at least 5 or higher [1]. The higher the number of imputations the more stable get the variability of the estimates [3]. Few authors’ recommend 50 or even more iterations, but they consider the number of at least 20 as appropriate [2-4]. For almost all imputed variables, the relative efficiency showed good values of 0.99, just two variables showed 0.97 and 0.98. However, the required minimum relative efficiency of 95 % [5] is maintained with the number of 20 iterations.

1. Jakobsen JC, Gluud C, Wetterslev J, Winkel P: **When and how should multiple imputation be used for handling missing data in randomised clinical trials - a practical guide with flowcharts**. *BMC medical research methodology* 2017, **17**(1):162-162.

2. Sterne JA, White IR, Carlin JB, Spratt M, Royston P, Kenward MG, Wood AM, Carpenter JR: **Multiple imputation for missing data in epidemiological and clinical research: potential and pitfalls**. *BMJ* 2009, **338**:b2393.

3. Horton NJ, Lipsitz SR: **Multiple imputation in practice: comparison of software packages for regression models with missing variables**. *The American Statistician* 2001, **55**(3):244-254.

4. Böwing-Schmalenbrock M, Jurczok A: **Multiple Imputation in der Praxis: ein sozialwissenschaftliches Anwendungsbeispiel**. 2011.

5. Berglund P, Heeringa SG: **Multiple imputation of missing data using SAS**: SAS Institute; 2014.

# Supplement: Further details diagnoses

Table 1: Details about the composition of the number of diagnoses

| N = | diagnoses | ICD-10 |
| --- | --- | --- |
| 104 | Total number of diagnoses of schizophrenia and schizoaffective disorder | F2x. |
| 48 | Total number of diagnoses of bipolar disorder | F3x. |
| 29 | Participants with two or three diagnoses (both diagnoses groups) | F2x./F3x. |
| 21 | Participants with a diagnosis in each of the two diagnoses groups | F2x. & F3x. |
| 10 | Participants with more than one diagnose in the schizophrenia and schizoaffective diagnosis group | F2x. |
| 3 | Participants with more than one diagnose in the bipolar diagnosis group | F3x. |
